# Young Adults’ Qualitative Self-Reports of Their Outcomes of Online Sexual Activities

**DOI:** 10.3390/ejihpe11020023

**Published:** 2021-03-30

**Authors:** Erin Leigh Courtice, Krystelle Shaughnessy, Kristin Blom, Yodit Asrat, Kristian Daneback, Nicola Döring, Christian Grov, E. Sandra Byers

**Affiliations:** 1 School of Psychology, University of Ottawa, Ottawa, ON K1N 6N5, Canada; ecour048@uottawa.ca; 2 Department of Social Work, University of Gothenburg, 405 30 Göteborg, Sweden; kristin.blom@socwork.gu.se (K.B.); kristian.daneback@socwork.gu.se (K.D.); 3 Department of Psychology, Trent University, Peterborough, ON K9J 7B8, Canada; yasrat@trentu.ca; 4 Institute of Media and Communication Science, Ilmenau University of Technology, Ehrenbergstraße 29, 98693 Ilmenau, Germany; Nicola.Doering@tu-ilmenau.de; 5 Department of Community Health and Social Sciences, CUNY Graduate School of Public Health and Health Policy, New York, NY 10027, USA; cgrov@sph.cuny.edu; 6 Department of Psychology, University of New Brunswick, Fredericton, NB E3B 5A3, Canada; byers@unb.ca

**Keywords:** online sexual activity, cybersexuality, cybersex, sexually explicit material online, online pornography

## Abstract

Online sexual activities (OSA) refer to Internet-based activities, behaviours, and materials that are sexual in nature. Many young adults engage in OSA, but report doing so infrequently. Most OSA outcome research has focused on negative effects of only some types of OSA (e.g., viewing pornography online). The goal of this study was to enhance knowledge on the range of OSA outcomes by qualitatively exploring young adults’ self-reported negative and positive outcomes from OSA experiences generally. University/College students from Canada (*n* = 246), Germany (*n* = 411), Sweden (*n* = 299), and the USA (*n* = 123) completed an online survey that included open-ended questions about “one of the most positive/negative effects that engaging in online sexual activities has had on your life”. More participants provided positive outcome responses than negative outcome responses. Qualitative analysis of the responses suggested a wide range of positive and negative outcome content that fit into seven bi-polar, higher-order themes: No Outcomes, Relationship Outcomes, Sexual Experience, Emotional Outcomes, Knowledge, Personal Outcomes, and Security. We found no variations in themes or their respective codes across the four countries. The findings suggests that researchers, educators, health care and psychology providers need to include multiple dimensions of positive and negative, personal and interpersonal, sexual and non-sexual OSA outcomes in their work

## 1. Introduction

The majority of young adults report engaging in one or more types of online sexual activity [1,2,3,4]. Online sexual activities (OSA) refer to Internet-based activities, behaviours, and materials that are sexual in nature [5]. There is a growing body of research on the prevalence and frequency of OSAs globally (e.g., Döring et al. [3]; Ballester-Arnal et al. [6]; Shaughnessy et al. [7]; Zheng and Zheng [8]). However, there has been less research on the outcomes of these activities—that is, the impact of people’s own OSA (whether use is infrequent or extensive) on people’s lives. Both the popular media as well as researchers have focused primarily on problematic outcomes (see Döring [1]; Kohut et al. [9]). This focus reflects assumptions that OSA detrimentally affects individuals and relationships. [9] As such, the instruments researchers used to assess OSA outcomes typically have contained a limited number of possible impacts—that is, they are biased toward negative outcomes [5,10,11,12,13]. As the outcomes contained in these measures are researcher-derived, rather than generated from participants, the extent to which they reflect people’s perceptions of the most important outcomes of their OSA is unknown. Thus, the purpose of this study was to explore young adults’ perceptions of both their negative and positive outcomes from engaging in a broad range of OSAs.

### 1.1. Perceived Positive and Negative Outcomes of Online Sexual Activities

Research on the perceived positive and negative outcomes of OSA is sparse, particularly for adult populations. In general, researchers have found that adults perceive more positive than negative outcomes to their OSA use and many perceive no negative outcomes at all. [5,10,13,14] In fact, Ranieri and colleagues [15] recently found no significant differences in psychological well-being between young adults who were intensive internet users and those who were cybersex users. These results stand in contrast to the common discourse about OSA outcomes that stresses harmful outcomes such as addictive usage patterns, sexual dysfunction, sexual victimization, sexual perpetration, or severe relationship conflict (e.g., Hermand et al. [16]; Ruvalcaba and Eaton [17]). However, OSA outcome research is limited by a focus on only a few OSA types (e.g., pornography or cybersex) and outcomes. For example, Kohut et al. [9] examined the perceived effects of pornography use (accessed online or offline) on the couple relationship (also Vaillancourt-Morel et al. [18] for a review). Similarly, Grov and colleagues [13] examined the perceived effects of viewing sexually explicit material, and a number of researchers have explored relationships between sexting (similar to cybersex, partner-seeking OSAs) and relational and/or sexual outcomes (e.g., Brodie et al. [19]; Dir and Cyders [20]; Drouin et al. [21]).

There are, however, many specific OSAs and these can be grouped into multiple types. For instance, Döring [1] identified six broad categories of OSA: sexual information (i.e., online exchange, consumption, or dissemination of sexual knowledge); sexual entertainment (i.e., watching or distributing sexually explicit material); sexual contact (i.e., sexual partner seeking, and technology-mediated sexual interactions; Courtice and Shaughnessy [22]); sexual minority communities online (e.g., lesbian, gay, bisexual, and queer or kink/fetish); sexual products online (i.e., browse, sell, or purchase sexual products); and, sexual commerce (i.e., offering or paying for online sexual services or online marketing for in-person/offline professional sexual services). Although these types of OSA are distinct, people who engage in one type of OSA are more likely to engage in other OSA types. [7,23] Given that many people engage in multiple types of OSAs, sometimes in the same time period (e.g., viewing pornography and sending sexually explicit messages), it is unlikely that people can separate the effects of one type of OSA from the effects of others. Thus, it is important to assess perceptions of the positive and negative outcomes of OSA experiences as a whole, rather than outcomes from one specific OSA or one type of OSA.

A second limitation to the research on outcomes of OSA is that most of these studies have used researcher-constructed, self-report, quantitative measures. There are few qualitative studies in which researchers attempt to examine OSA outcomes from a participant-driven, or lived experience, perspective. Indeed, Kohut et al. [9] pointed to the need for participant-driven, “bottom-up” research to inform future studies on pornography outcomes whether viewing occurs on- or offline. Additionally, researcher-constructed measures vary in their number of items and most have unknown psychometric properties. For example, for the most part, researchers have not ensured that these measures have content validity—that is, include the full range of positive and negative outcomes that people experience. Moreover, researchers generally assigned a valance (positive or negative) to each outcome they assessed. Some of the included outcomes are obviously positive (e.g., improved understanding of sexual preferences) or negative (e.g., spending too much money online). However, others are ambiguous (e.g., increased frequency of masturbation) and may represent a positive outcome for some participants, a negative outcome for others, and a neutral outcome for still others (see Hald and colleagues [24,25]). In sum, use of researcher-driven, self-report measures significantly limits knowledge about the degree to which OSAs have negative, positive, neutral, or varying outcomes related to a wide range of life domains.

Three qualitative studies have shed light on people’s lived experiences. McKie and colleagues [26,27] conducted two focus group studies that examined young gay men’s perceptions of the benefits and challenges of technology use in and for sexual and romantic relationships. Their thematic analyses suggested that these men perceived both negative and positive outcomes of technology use. That is, participants reported that technology use created challenges and opportunities in their romantic and sexual relationship development. The themes also suggest that technology facilitated finding and choosing sexual partners, and contributed to sexual identity development. McKie and colleagues [26,27] examined positive and negative themes separately; however, their findings seemed to indicate bi-polar (that is, having two opposing ends or dual ‘poles’) outcome themes. The extent to which these results would generalize to women and/or people who identify as heterosexual also is not known.

Kohut et al. [9] examined the perceived outcomes of pornography use on the couple relationship in a large sample (*N* = 430) of heterosexual individuals. They identified 66 themes and subthemes from participant responses. A substantial number of participants identified positive impacts on them, their partner, and their relationship (e.g., improved sexual communication, greater sexual experimentation, enhanced sexual comfort). Fewer participants identified negative effects (e.g., unrealistic expectations, decreased interest in the partner, increased insecurity). Indeed, the most common theme was a denial of any negative effects (referenced in 15.7% of the responses). The extent to which these findings are generalizable to people with a same-sex partner, people who are not currently in a relationship, or other types of OSA are unknown.

### 1.2. Current Study

The goal of this study was to enhance our understanding of the range of outcomes of engaging in OSA by exploring young people’s perceptions of their lived positive and/or negative outcomes of their own OSA. We did this in two ways. Our first research objective was to identify a wide range of positive and negative outcomes that people identify as resulting from their OSA. Our second objective was to identify themes, within or across valences, that capture the range of outcomes more concisely. To do so, we collected responses to open-ended questions about positive and negative outcomes of university/college students’ OSAs in four countries (Canada, Sweden, Germany, and USA). We expected this approach would capture a range of outcomes that are common to Western countries and any that are culturally specific. Culture can have a profound influence on sexual behaviour through sexual socialization to country and cultural values [28,29,30,31]. Thus, throughout the qualitative analytic process, we explored whether differences between countries emerged in the codes and themes. We chose to study young adults because research suggests that this age group is likely to engage in multiple types of OSA (e.g., Döring et al. [3]; Shaughnessy et al. [7]).

## 2. Methods

### 2.1. Participants and Procedures

We recruited 2720 students aged 18 and older, from four higher education institutions (one each in the United States, Canada, Sweden, and Germany) in 2012, to participate in an online survey about their attitudes, experiences, and outcomes of OSAs. The U.S. institution was situated in the Northeastern U.S.; the Canadian institution was in Eastern Canada; the Swedish institution was in Western Sweden; and the German institution in Central Germany. We chose these countries because some of the authors were residing in and affiliated with academic institutions in these locations at the time of data collection. We recruited participants by using university list-serves, flyers, undergraduate study research pools, and word of mouth. The recruitment materials (which were translated into the appropriate language for each country) indicated that the study was about a range of online behaviors, including online dating, chatting, cybersex, and pornography. Findings related to OSA experiences were published in a separate article [3].

The current sample consisted of 1090 participants (40.1%) who indicated that they had engaged in at least one OSA on the OSA Experiences measure (see measures) and provided written answers to the questions about positive and/or negative outcomes: 125 from the U.S, 248 from Canada, 304 from Sweden, and 413 from Germany. Of these, 582 identified as women, 497 identified as men, and 11 as either transgender (MtoF = 3) or an ‘other’ gender (e.g., non-binary). On average, participants were 24.5 years old (*SD* = 5.26). Most reported being in a romantic relationship (58.5%) at the time of the survey. Some participants (*n* = 56) did not report their sexual identity; of those who did, most identified as heterosexual (76.1%) and the remainder with a range of sexual minority identities that differed somewhat across languages.

Each university/country had its own survey link to track participant location and to present participants with the survey in the dominant language of their country of residence. After providing informed consent, participants were directed to the online survey that took approximately 10–15 min to complete. The survey contained a background questionnaire, OSA experiences questionnaire, the open-ended OSA outcome questions used in this manuscript, and other self-report measures related to sexuality but not relevant for the present study. All study material were prepared in English and translated, then back-translated, by German and Swedish first-language researchers. Participants received debriefing information on a webpage at the end of the survey. Some participants in the Canadian sample received course credit towards an introductory psychology course for participating in this study; the other participants were not remunerated. The Institutional Review Board/Research Ethics Board at the U.S. and Canadian institutions approved this study; separate approval was not required for the German and Swedish locations at the time because of different international regulations regarding the use of human subjects in multi-site research. Indeed, the European Union’s General Data Protection Regulation (GDPR) was implemented in 2018, after we collected data for this study. However, we did adhere to the ethical procedures outlined by the GDPR: we did not collect or store identifying information (e.g., names or IP addresses), required informed consent from participants, and this study had no potential for harm.

### 2.2. Measures

#### 2.2.1. Sociodemographics

Participants responded to questions regarding their sociodemographic characteristics including gender, country (Canada, Germany, Sweden, U.S.), age (in years), sexual identity, and relationship status. Both sexual orientation and gender were assessed using closed questions for which participants selected the option from a list of culturally appropriate options that best applied to them. An option of ‘other’ was available for those who did not see their sexual or gender identity represented; if participants selected ‘other’, they were asked to specify.

#### 2.2.2. OSA Experiences

Participants indicated whether they had engaged in each of 24 OSA experiences in their lifetime. All six categories of OSA as described by Döring [1] were assessed. Readers interested in the prevalence and frequency of each OSA are encouraged to read the manuscript using the same dataset as the current study but focused on OSA experiences [3].

#### 2.2.3. Outcomes of OSA

Participants who indicated that they had engaged in one or more OSAs were asked to complete two open-ended items that assessed their perceptions of the positive and negative outcomes of their OSA experiences. Participants were provided the following information: “Online sexual activities can have different positive and negative effects on people. The following questions ask about the effects of engaging in online sexual activities on you”. We then asked them in separate questions to: “Describe one of the most [positive/negative] effects that engaging in online sexual activities has had on your life” in a text box that did not have a word limit.

### 2.3. Thematic Analysis and Qualitative Coding

We used an inductive thematic analysis approach to examine participants’ responses to the open-ended OSA outcomes questions. Thematic analysis refers to a cluster of qualitative methodologies, focused on identifying patterns in data [32]. In an inductive thematic analysis approach, the data itself directs the development of codes [33,34]. To address our first research objective, we identified codes related to perceived positive outcomes separately from the perceived negative outcomes. We aimed to be comprehensive in our codes to capture as many perceived outcomes as possible. We subsequently examined the codes for common themes within and across the positive and negative outcomes to address the second research objective. Thus, we had two levels of analysis—the lowest being codes that focused on content and the highest being themes that captured a range of ideas or meanings within and across codes together.

Our specific qualitative data coding procedure was as follows. First, two North American researchers examined 100 randomly selected responses from the Canadian and U.S. datasets. These researchers independently generated codes and then compared their coding structures with each other to agree upon a final set of codes. This process resulted in 23 positive outcome codes and 22 negative outcome codes. Second, two independent bilingual researchers in Sweden and two in Germany evaluated 100 randomly selected responses in those respective datasets for fit with the initial coding scheme. This process confirmed that the codes generated from the North American data fit with the German and Swedish data and that no new codes were needed. Third, all responses from each of the four countries were independently coded: two North American researchers coded the data from Canada and the U.S., two German researchers coded the data from Germany, and two Swedish researchers coded the data from Sweden. In cases where a participant discussed multiple outcomes, all appropriate codes were applied (*overlap* or *multiple coding*; Saldana [35]). No new codes were identified at this stage. Inter-rater reliability (Boyatzis [36]) was high for all the codes in all four countries: kappa values ranged from κ = 0.65 to 1.0. Disagreements were resolved through discussion and consensus.

Next, two authors looked for and discussed higher-order patterns in the positive outcome and negative outcome codes separately. The goal of discussions was to propose an organization of codes that meaningfully depicted the range of responses [37]. The second author then examined the proposed thematic structure. They noted that the higher-order themes appeared to be bi-polar in nature—meaning that the same themes were evident in both positive and negative OSA outcome codes, such that they contained two opposing ends or dual ‘poles’. The codes were reorganized to represent the final six higher-order bi-polar themes and presented to the second and last author. After considering their feedback, the first and second author used NVIVO 11 and Google translate (to translate German and Swedish responses to English) to recode all of the data into the six themes directly to ensure that the positive and negative codes fell into the themes as proposed. All translations were reviewed by bilingual researchers (fluent in both English and German or English and Swedish, respectively) to verify the accuracy.

## 3. Results

### 3.1. Descriptive Aspects of the Responses

Responses ranged from single words to multiple, descriptive sentences. Some responses reflected only one code and theme; others reflected multiple codes and themes. Thus, there were more data components in responses than there were number of participants in the sample. Most participants responded to both the positive and negative OSA outcomes question. However, there were many who responded to only the positive OSA outcomes question, and a few who only responded to the negative OSA outcomes. We also observed that some responses reflected outcomes that occurred because of a partner’s OSA experience rather than their own experience. As the questions specifically focused on outcomes of one’s own experience, we excluded these units (either response in whole, or portion of response) from the analysis.

All codes and themes appeared in each country; there were no codes or themes unique to or absent from any one country. Thus, our descriptions of codes and themes apply equally to Canadian, U.S., German, and Swedish responses. Due to the range in codes, coding positive and negative responses separately, over-coding, and collapsing codes into themes across rather than within the positive and negative outcomes, we did not quantify the frequency with which a code or theme occurred. However, no one code or theme reflected a majority of responses for either the positive or negative outcomes. In the tables and in text, we provide examples of codes and themes in participant responses; to ensure representation of participants from all countries, we used the Google translated versions of German and Swedish responses (verified by a German and Swedish author).

### 3.2. Positive and Negative Content Codes

Participant responses reflected a wide range of positive and negative outcome codes, examined within the positive and negative responses separately. Specifically, our analyses yielded 14 positive outcome codes and 15 negative outcome codes. We list the codes, a brief description of each, and a sample response in Table 1. Some participants wrote that they had experienced no positive and/or no outcomes. We coded these as *No positive outcome* (e.g., “There are no positive effects”; *n* = 151, 13.8%) and *No negative outcome* (e.g., “I have not experienced any negative outcomes”; *n* = 307, 27.8%) to be consistent with previous research (e.g., Kohut et al. [9]). This code did not include blank responses—which were excluded previously from the response set. Notably, 119 participants responded with *No positive outcome* and *No negative outcome* explicitly (not as blank entries, e.g., “There isn’t any that I can think of that spring to mind”.).

### 3.3. Higher-Order Bi-Polar Themes

**Figure 1:**
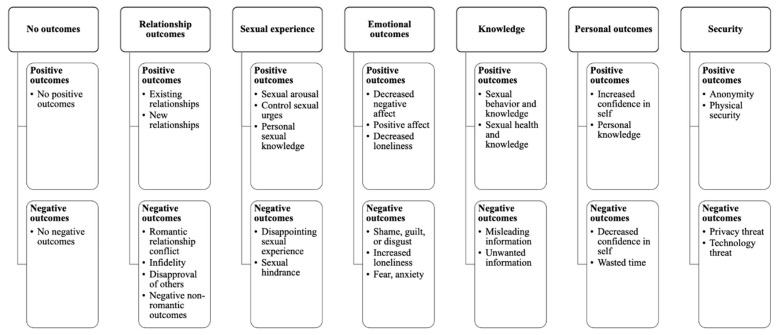
Bi-polar themes and their associated positive and negative outcome themes.

Our qualitative analysis resulted in seven higher-order bi-polar themes that captured positive codes and negative outcome codes together: *No Outcomes, Relationship Outcomes, Sexual Experience, Emotional Outcomes, Knowledge, Personal Outcomes, and Security.* Themes are listed in Table 1 along with the positive and negative codes categorized within each, and sample responses fitting each code that also demonstrate the themes. A visual summary of the themes and codes is presented in Figure 1.

The theme *No Outcomes* captures responses in which participants explicitly stated having not experienced any positive and/or any negative outcomes from their OSAs.

In terms of the theme *Relationship Outcomes,* participants described both positive and negative ways that their OSA impacted romantic, sexual, peer, and family relationships. Many participants described positive relational outcomes involving benefits for existing relationships (e.g, “spicing up a long-term relationship”) or developing new relationships (e.g., “I’ve met the man I’m going to marry”). Many of these responses also reflected relational sexuality specifically. For some participants, the benefit of OSA was in finding sexual partners (e.g.,“As a gay man, it is an everyday thing to seek sex or partners online”; translated from Swedish), establishing a relationship (e.g., “I met my fiancé on a dating website”), and/or maintaining sexual intimacy in a long-distance relationship (e.g., “Lets you be intimate with a committed partner when in a long distance relationship”). Still others noted finding people with shared interests who may or may not become sexual partners, such as “Chance to meet like-minded people and have a good time” and “Getting to know people through whom one can talk openly about certain aspects of sexuality, especially BDSM, and thus increase safety aspects in BDSM games” (translated from German). In terms of negative outcomes, some participants perceived that OSA had affected their relationship negatively including a negative impact on their enjoyment of sex with a partner or that OSA interfered with their offline sexual activities. Some linked their OSA to a perceived difficulty remaining “faithful” to their partner or having cheated on their partner (e.g., “I had cybersex with an unknown man which gives me a bad conscience before my husband”; translated from Swedish). Finally, some participants described negative relationship outcomes of their OSA use for relationships with people they were not in a committed romantic relationship with (e.g., “When I was 18 a younger friend of mine insisted on trying to send my sexually suggestive photographs of herself, which would I subsequently ignore. This upset her.”.) or parents (e.g.,“ embarrassing reactions from parents”.).

The *Sexual Experience* theme captures participants’ responses that indicated that their OSA(s) had contributed positively or negatively to their sexual behaviours, activities, arousal, response, or sexual interests in some way. On the positive end, some participants described experiencing sexual arousal, orgasm, sexual pleasure, or sexual enjoyment from their OSAs. Some participants reported that their OSAs allowed them to satisfy sexual desires that they could not or did not want to satisfy in offline, in-person ways; sometimes these desires were wanted and sometimes they were identified as unwanted (e.g., “Self pleasure and explore fantasies”, “sometimes want “regular sex” but it can be a bit boring”.; translated from German). Participants also indicated that their OSA experiences helped them to learn about or develop their own sexual likes or dislikes (e.g., “I have learned about what turns me on”). On the negative end, some participants found their OSAs disappointing in that they did not “meet expectations”, were “not as fulfilling as the real thing”, or were “less stimulating than real-life sex”. Others indicated or suspected that their OSAs interfered with or hindered their offline sexual activities in some way including “decreased sexual desire”, “sexual numbness”, and “lack of fulfillment”. Some responses suggested that OSAs interfered with sexual aspects of relationships specifically (e.g., “causing you to have less sexual motivation with your partner”); whereas, other responses did not clearly link interference to relationships (e.g., “normal sexual acts are mundane to me now and no longer stimulating”).

A number of participants indicated that their OSA experience(s) had altered their emotional state in some way, for better or worse. We called this theme *Emotional Outcomes* to capture all responses that related to prompting positive or negative emotions or affective states, or alleviating negative emotions or affective states. For instance, as a positive outcome, some participants indicated that their OSA resulted in a reduction in negative affect or physiological sensations related to emotional experiences including stress, tension, boredom, or loneliness. Some participants wrote that their OSA resulted in increased positive emotions such as relaxation, calm, entertainment, or enjoyment (without specifying that these were sexual). Some indicated both increased positive and decreased negative emotions at once such as: “Relaxation through casual sexual stimulation…”. (Translated from German). As a negative outcome, participants indicated that OSA resulted in emotions such as shame, guilt, disgust, or loneliness generally or with respect to sexuality specifically (e.g., “I felt dirty afterwards”; “I often feel ashamed looking up erotic pictures online”). Some participants also indicated that their OSA prompted fear, worry, or embarrassment especially of being caught (e.g., “It is very embarrassing to be caught doing this”.), or of their content (messages, pictures) being accessed, kept, or shared by others (e.g., “Fear of the video or conversation being distributed or stolen because it is online”). The latter also reflected the Security theme (described below).

Some participants identified changes in their *Knowledge* as an outcome of their OSA experience(s). Some participants described information acquisition as a positive outcome in itself (e.g., “Gain knowledge and experience”; “Learned some new information and different things”). Others specified that new or expanded knowledge was positive because it improved their understanding of sexual behaviours, techniques, and/or sexual health. For example, (e.g., “Found a nice and new position that me and my boyfriend liked”; translated from Swedish) and (e.g., “Provide information on what counters birth control”). A few participants described information acquisition as negative, often noting that they were exposed to false, misleading, or unwanted information, or because it resulted in negative views of others. For example, one participant encountered “Some misguided ideas about penis size, what constitutes normal sex, etc.”; another noted, “It may have created some form of fear, as some images found are not a true illustration of what happens in reality in respect to the level of partner’s comfort”.

Some participants indicated that their OSA experience(s) had impacted their thoughts, feelings, and comfort with themselves or their routines, termed *Personal Outcomes*. Positive *Personal Outcomes* included improved self-confidence or self-esteem with respect to their sexuality/attractiveness (e.g., “Increase self-esteem”; “It has made me more confident and comfortable about my sexuality”), and personal knowledge of their sexual self (e.g., “a better understanding of [their] sexual and sensual side that can’t be taught or was not taught in school”). In contrast, some participants noted that their OSA contributed to a decrease in their self-confidence or self-esteem, at times linked to their body image, feelings of attractiveness, or sexual abilities. Other responses suggested negative perceptions of the value of OSA. For example, a few participants believed that they had “wasted time”, or “spent lots of money”; some suggested that their time spent on OSA was to the detriment of other activities such as “In doing so, I lose a lot of time that I could use to do other things…”. (Translated from German), or “I waste a lot of time masturbating and use it as a means of procrastinating”.

A few participants indicated that their OSA(s) had impacted their sense of *Security*, particularly in terms of safety and privacy in sexual and cyber/technological realms. Positive outcomes related to *Security* included anonymity—or the ability to hide one’s identity while using OSAs (e.g., “fast, anonymous, …”). Some participants indicated that physical security was a positive outcome of OSAs (e.g., “It allows me to explore sexual fantasies without engaging in behaviours that could be harmful to my physical and mental health”.), including a lack of potential sexual health consequences (e.g., “not having to worry about diseases”). For some, anonymity was associated with reduced fear or anxiety, as in “It can be completely anonymous. You don’t have to worry much about people you know finding out”. For others, anonymity was important because it provided them with a sense of greater freedom to engage in sexual exploration than they would experience offline (e.g., “You are anonymous and can be anybody and change one’s preferences thereafter (try out different things that you may not normally have done)”; translated from Swedish). Some participants described negative *Security* outcomes from privacy threats and technological threats. Privacy threats were focused mostly on concerns that other people would or did share the sexual material that the participant had created such as “The threat that someone may post what you’ve said or blackmail you with your picture etc.”; these also involved concerns of being exploited, or harm to reputation from other people learning about their OSAs such as this participant who wrote “At the risk of sounding conceited, my social status and reputation are always a serious concern”. Participants also identified a number of technological threats including “the spam on my computer”, “viruses”, “huge amounts of cookies”, and advertisements (e.g., “a lot of advertisements and other things that can make one both annoyed and angry”).

## 4. Discussion

The purpose of this study was to explore young adults’ perceptions of both negative and positive outcomes that resulted from engaging in a broad range of OSAs. The results revealed the wide range of individual outcomes that young people connect to their general OSA use, as well as the primary domains that these outcomes fall into: Relationship Outcomes, Sexual Experience, Emotional Outcomes, Knowledge, Personal Outcomes, and Security. In so doing, these results extend past literature on OSA outcomes that has suffered from a narrow focus on specific OSAs, assumptions about the valence of a particular outcome, and a bias towards assessing negative outcomes. Furthermore, the results suggest that OSA outcomes are similar for young people, at least across Western countries.

### 4.1. Positive, Negative, or No Outcome?

Most of our participants responded to both the question regarding negative and the question regarding positive outcomes of OSA. That is, they were able to identify both positive and negative outcomes from their OSA use. Nonetheless, many participants did not do so. Some explicitly reported that they did not experience any positive or negative outcomes from their OSA use. This finding is consistent with Kohut and colleagues [9] who reported that approximately 16% of their participants indicated that pornography use had no negative effects on their relationship. Others perceived, identified, and elaborated on positive but not negative outcomes of their OSA. In contrast, few participants identified negative but not positive outcomes. These results extend past findings based on researcher-derived quantitative outcome measures focused on specific types of OSA that found people report more positive than negative outcomes of their use (Albright [10]; Döring and Mohseni [14]; Grov et al. [13]; Ranieri et al. [15]; Shaughnessy et al. [5]). Third, almost all participants who reported a negative outcome also reported a positive outcome; only a few participants gave answers to the negative outcome only question, and many (28%) gave an explicit statement of no negative outcomes as their response (compared to 14% for explicitly no positive outcome). Taken together, these results suggest that to fully understand the outcomes of OSA, it is important for researchers to use measures that do not inherently problematize OSA experiences and instead examine a range of positive, negative, and neutral outcomes. Our results suggest that for many people, their OSA experiences may be inconsequential to their life—providing no notable outcomes at all, or positive and negative outcomes that balance each other out or are short lived. Our findings have implications for, and are aligned with, the positive sexuality framework (Williams at al. [38]) and the positive technology framework (Riva et al. [39])—perspectives originated from the positive psychology approach (Seligman and Csikszentmihalyi [40]). For example, Döring and Mohseni [14] used these positive sexuality and technology frameworks to explain their findings that people reported more positive than negative outcomes related to their experiences with OSAs and sexting. As such, these results contribute to the growing body of evidence that OSA discussions in research, education, clinical, and media might be focused too much on negative outcomes.

### 4.2. Variability in OSA Outomes and Themes

Our results paint a nuanced and multidimensional picture of people’s perceptions of the outcomes of their OSA. By identifying both content codes in outcomes and overarching themes, the results reveal variability in the specific and broader outcomes that people experience. The six bi-polar themes that characterized the outcome content (the seventh theme was no outcomes) provide a more parsimonious way of assessing and understanding outcomes of OSA. This finding extends the literature in a couple of ways. First, they clearly point to the likelihood of positive and negative outcomes on the same dimension, stemming from the same types of OSA experiences. That is, the findings suggest that one person’s OSA experience leading to a positive outcome may be another person’s similar experience but leading to a negative outcome. For example, engaging in cybersex might decrease loneliness for one person, but increase loneliness for another person. Second, some people may experience positive and negative outcomes on the same or different dimensions of their lives from their OSA experience. For example, watching pornography online may both alleviate stress and tension (positive Emotional Outcome) but prompt worry (negative Emotional Outcome) about others finding out (negative Relationship Outcome). Thus, researchers need to consider the potential for positive, negative, and mixed outcomes of OSA on multiple life dimensions.

Previously, researchers have implied that OSA outcomes are uni-valenced, rather than bi-polar. For example, McKie and colleagues [26,27] findings seemed to suggest that OSA outcomes could be bi-polar, but they did not analyze their results in this way. Kohut and colleagues [9] identified a comprehensive list of 66 themes and subthemes of people’s OSA outcomes. However, some of those reported also appeared very similar to each other (e.g., “Jealousy” or “Envy”, “Anger Resentment” or “Conflict”, and “Disgusted” or “Disturbed with Partner’s Use”). Others are consistent with the bi-polar concept. For example, Kohut and colleagues found that some participants reported watching pornography as positive because it “increased arousal response”, and others perceived the negative outcome of “decreased arousal response”. Both of these themes would be captured under our bi-polar theme, Sexual Experience. Indeed, bi-polar themes provide a more parsimonious way of capturing, and thus assessing outcomes of OSA.

Second, the results of the content coding point to the wide range in the potential impacts of OSA. For example, these results suggest that although two individuals may experience a positive or a negative impact on a particular dimension of their life as represented by the themes (e.g., their emotions), they may differ in the specific aspect of the dimension that is affected. Indeed, each of the six bi-polar themes contain many different contents on the positive and negative ends; for example, participants identified two positive outcomes (anonymity and physical security) and two negative outcomes (privacy threat and technology threat) related to Security. In addition, although some of the specific outcomes appear to be temporary or short-lived (e.g., stress and tension relief/relaxation, sexual arousal, entertainment/fun); others appear to reflect longer terms and perhaps more substantial outcomes (e.g., new relationships, personal knowledge). Furthermore, people identified that OSA experiences can affect them both personally (e.g., sexual health and knowledge, emotional outcomes, increased or decreased self-confidence, physical security) and interpersonally (e.g., romantic and/or sexual relationships). Although many OSA outcomes related to sexuality, participants identified positive and negative outcomes that extended beyond their personal sexual lives such as time wasting, technology threats, decreased loneliness and isolation, and increased or decreased self-confidence. In combination, these findings suggest that some outcomes of OSA use might satisfy immediate needs, whereas others might relate to greater self or relational actualization. Future research on motivations for OSAs, and how these predict outcomes, is needed.

Some of our themes and content codes represent outcomes that have rarely or never been included in researcher-derived OSA outcome measures or identified in qualitative research. For example, our results highlighted young adults’ perceptions that their Security was impacted by their OSA in both positive and negative ways. Despite theoretical descriptions that align OSAs with providing access to sexual stimuli and activities while simultaneously protecting physical security and anonymity, very few researchers have attended to the specifically technological aspects of people’s OSA experiences. For example, McKie and colleagues’ [26] findings included a discussion of the lessened risk of online communication, however the *security* afforded by technology (and other specific technological components of OSA) was not identified as a theme in their results. The current results point to the importance of assessing as threats of others gaining access to self-created sexual material, or information on one’s OSA and using that to exploit, harm, or hurt the person as two examples of possible outcomes. Indeed, as people have become more aware of technological threats (e.g., privacy and data sharing web breaches), concerns about and experiences with such threats may increase. Our participants also identified maintaining sexual connections with a partner an important outcome of OSA. Yet, few researchers have examined the outcomes of OSA in long-distance relationships (Goldsmith and Byers [41] for an exception), despite the likelihood that some forms of OSA may be particularly common in this context. These findings indicate that Hald and colleagues’ [25] approach to assessing extent and participants’ perceived valence of outcomes could be expanded to assess a broader range of OSA (not just sexually explicit material) outcomes.

## 5. Limitations, Future Directions, and Implications

The results of our qualitative analyses with written responses provided by university students in four Western countries provide novel information on OSA outcomes. However, this study has some limitations. First, our data came from a convenience sample of undergraduate university students residing in Western nations; the majority identified as heterosexual and cisgender. It is possible that the relatively homogenous demographic aspects of our sample obscured differences that might otherwise exist between the different countries. Second, although our findings present a novel contribution to the literature on OSAs, our data was collected in 2012. Indeed, the world is rapidly evolving in relation to people’s use of the internet for OSAs. For example, researchers have suggested that the current COVID-19 pandemic may have changed people’s prevalence and patterns of OSA use—at least in the short-term [42,43,44]. Third, the written responses provided by our participants varied in detail, most being relatively concise. Using focused written responses are beneficial in that they allow researchers to easily pull out key themes and ideas. However, participant response may be more nuanced than suggested by their entries—a point that would only arise with interview or focus group style follow up. Fourth, in this study, we asked participants to identify *one of the most* positive and *one of the most* negative outcomes. This suggests that all of the outcomes listed were meaningful to participants. Nonetheless, research that assesses the magnitude of the positive and negative impacts of outcomes within each of the themes, and possibly codes, could shed light on the relative importance of each domain to people’s lives. Fifth, we did not examine whether specific codes or categories arose because of experience with particular OSA subtypes (e.g., outcomes related to pornography use—a subtype of OSA). We did so because many young adults report more than one OSA experience that crosses subtypes of OSAs [7]. Our examination of outcomes of OSA generally allowed us to learn about positive and negative outcomes that likely span OSA subtypes. However, we cannot say whether particular outcomes occur in the context of specific OSAs. Even though we surveyed a relatively large sample from the population of college students, some particularly severe but seldom outcomes (e.g., clinical symptoms; criminal behavior) are underrepresented.

Our findings raise a number of future directions for OSA researchers. First, the findings require replication and extension in non-Western, sexual and gender identity diverse samples, and with older adults. Additionally, people who are in different life stages, regardless of age, may perceive outcomes of OSA that are not well represented in our findings (e.g., transition to parenthood or to widowhood may raise novel OSA outcomes). Our findings and those of other qualitative research (e.g., Kohut et al. [9]) emphasize the importance of understanding outcomes from the perspective of the person engaged in the experience—in our case, the OSA user—prior to constructing quantitative research to examine prevalence or predictors of outcomes. Future research should use our findings to construct content valid self-report measures, with strong psychometric properties prior to using these to quantitatively examine OSA outcomes. Such measures should cover multiple sexual and non-sexual life domains, focus on personal and interpersonal outcomes, and allow participants to report positive and negative valenced outcomes on the same dimension. This measure development work will greatly contribute to a needed body of valid research on the nuanced variability in people’s OSA experiences and outcomes. Furthermore, it is important for researchers to better understand what drives, or motivates, peoples’ OSA experience, particularly in light of continued use when people experience negative outcomes.

Our results also revealed information relevant to improving education and health care practice related to OSA experience. Specifically, our findings suggest that more people might experience positive outcomes compared to negative outcomes from their OSA experiences. Indeed, our findings extend others in highlighting that people tend to perceive more positive and fewer negative outcomes of their OSAs. Moreover, the positive outcomes likely benefit people in many more ways than educators and service providers currently ask about or include in discussions and materials. These positive outcomes not only need to be acknowledged, but also used to improve harm reduction approaches to OSA education and treatment, and to create positive technology and sexuality programming (e.g., sex education curriculums, pornography and media literacy programs). Researchers have suggested that the COVID-19 pandemic has provided educators and health care providers with an opportunity to recommend OSAs as a public health measure (Döring [43]); indeed, this messaging could persist in non-pandemic times. For instance, health care providers could recommend OSAs as a sexual health intervention for those seeking communities of support, additional information, exploring legal and consensual sexual interest, desires, and relationships in a relatively safe space. Furthermore, we identified OSA outcomes beyond those related to people’s sexuality and romantic relationships; for example, participants identified both negative and positive outcomes related to security, such as privacy threats and enhanced physical security. Educators and health care providers need to ask about, educate on, and discuss OSA outcomes on many life dimensions—particularly non-sexuality specific areas—that are not obviously connected to the sexual identity or sexual stimuli content of OSAs. Indeed, health care providers may seek to incorporate questions about online safety with patients that engage in OSAs, and educators may need to include discussions about protecting one’s privacy when engaging in OSAs.

## 6. Conclusions

To our knowledge, this is the first international, qualitative study focused on people’s self-described positive and negative OSA outcomes. Our findings contribute novel information by suggesting that content of young adults’ perceived OSA outcomes fit a bi-polar structure, with negative and positive content falling into six core themes. Some of these themes, such as security, are not prevalent in OSA research. Overall, these findings contribute to the research demonstrating that people experience positive and negative outcomes (not one or the other) from their OSAs. They also point to the need for more research, education, and psychological interventions that balance the likely beneficial and potentially detrimental outcomes of OSA generally. The findings from this study add to those on pornography or technology generally, by emphasizing the importance of understanding outcomes from the perspective of the person engaged in the experience—in our case, people who engage in OSAs.

## Figures and Tables

**Table 1:** Description and examples of positive and negative outcome codes and their associated bi-polar theme.

Associated Bi-Polar Theme		Positive Outcomes			Negative Outcomes		
Theme Name	Brief Description	Positive Codes	Brief Description	Characteristic Quote	Negative Codes	Brief Description	Characteristic Quote
No outcomes	Captures responses in which participants explicitly stated having not experienced any positive and/or any negative outcomes from their OSAs	No positive outcomes	Explicitly wrote that they had not experienced any positive outcomes.	“I did not feel anything positive” ^b^	No negative outcomes	Explicitly wrote that they had not experienced any negative outcomes.	“I have not had any negative effects from engaging in online sexual activities”.
Relationship Outcomes	The ways that participants’ OSA experiences had an effect on their interpersonal relationships, romantic, sexual, or other.	Existing relationships New relationships	Benefits for existing relationships such as facilitating communication about desires, maintaining sexual and intimate connections in long-distance relationships. Involving the development of new relationships, such as finding new sexual partners or forging new friendships.	“Sometimes watching porn with my partner can open up the conversation about our own personal desires or fantasies”. “I met other young women with similar sex-positive attitudes to my own”.	Romantic relationship conflict Infidelity Disapproval of others Negative non-romantic outcomes	Experienced negative relational outcomes, including reduced enjoyment of sex with a partner, conflict in an existing romantic relationship, or conflict in a non-romantic relationship. Difficulties remaining “faithful” to their partner, or having cheated on their partner. “Looked down upon by others” Experiencing a negative relationship outcome with someone they were not in a committed romantic relationship with.	“negative reaction of my partner to my consumption of stimulating videos”. ^a^ “Since it is so easy to access it is much easier to discreetly cheat on a partner. I have a very hard time being faithful and online infidelity is the easiest to access and hide from a partner”. “Online communication differs significantly from offline communication. Said acquaintance doesn’t talk to me like that in real life. I found that uncomfortable and confusing”. ^a^
Sexual Experience	The ways that participants’ OSA experiences had contributed to their overall experience of/with sexuality.	Sexual arousal Control sexual urges Personal sexual knowledge	Experiencing sexual arousal, orgasm, sexual pleasure, or sexual enjoyment from their OSAs. Allowed them to satisfy sexual desires that they could not or did not want to satisfy in offline (in-person) ways; sometimes these desires were wanted or unwanted. Participants reported positive outcomes that that their OSA experiences helped them to learn about or develop their own sexual likes or dislikes.	“Supportive effect of stimulating videos for masturbation”. ^a^ “It is always an option for release, which helps me sleep and prevents me from being troubled by sexual thoughts”. “sexual stimulation, satisfaction, information about intimate questions about one’s own body/one’s own sexuality”.^a^	Disappoin- ting sexual experience Sexual hindrance	Reported their OSA experience to be disappointing or unfulfilling. OSA experiences interfered with or hindered their offline sexual activities.	“It was just sex that I thought I needed/wanted at the time. I never felt fulfilled or found a potential mate from these experiences”. “Sometimes the pornography has been so arousing that I have not been able to control my ejaculation, which may have led to a very short intercourse with my girlfriend, or a quick ejaculation during masturbation alone”. ^b^
Emotional Outcomes	The ways that participants’ OSA experience(s) altered their emotional state in some way.	Decreased negative affect Positive affect Decreased loneliness	Reducing negative emotions and/or their associated physiological sensations. Increased positive emotions such as relaxation, calm, entertainment, or enjoyment. Alleviating or declined sense of loneliness, and/or feeling connected with others.	“It helps you relax in times of high stress”. “Sometimes I’ve felt lonely so it’s just one other means of connecting with another person”	Shame, guilt, or disgust Increased loneliness Fear, anxiety	Prompted negative emotions such as shame, guilt, disgust. Feeling increased loneliness. Experiencing fear, worry, or embarrassment especially of being caught or of their content (messages, pictures) being accessed, kept, or shared by others.	“Feeling occasional guilt, shame, or regret”. W “Make me worry that I’ll never actually get a real girlfriend or sexual partner”. “Fear of someone else seeing or reading”. “Have considered that images can be misused. However, I have never posted pictures with my face on, but the thought has still struck me that those pictures are still somewhere out there”. ^b^
Knowledge	The ways that participants’ OSA experience(s) had contributed to their acquisition of information.	Sexual behavior and knowledge Sexual health and knowledge	Improved understanding of sexual behaviours. Related to an improved understanding of sexual health and sexual knowledge generally.	“Watching pornography has taught me sexual positions that I would not have thought of, which in turn has bettered my sex life”. “I have received information about contraceptives that suited me better than before, and I have been told that not only have I been in the same seat when it comes to partners’ sexual activities”. ^b^	Misleading information Unwanted information	Exposure to information that was false or misleading. Exposure to information that they did not want to learn.	“Some mis-guided ideas about penis size, what constitutes normal sex, etc. A bit misleading for teenage boys who have no sexual experience”. “I haven’t had a negative effect from online sexual activities beside seeing disgusting sexual acts that grossed me out”.
Personal Outcomes	The ways that participants’ OSA experiences had altered their views of themselves.	Increased confidence in self Personal knowledge	Related to an improved self-confidence or self-esteem with respect to their sexuality and/or appearance. Enhanced personal knowledge related to their sexuality.	“Self-confidence increases over one’s own body”. ^b^ “Learning more about my own sexuality and my sexual preferences”	Decreased confidence in self Wasted time	Related to a decrease in their self-confidence or self-esteem with respect to their sexuality and/or appearance. Negative perceptions of the time or energy spent in OSA.	“I can see my body on webcam, and I do not like it because I think my body is unattractive”. “Loss of time due to excessive pornography consumption”. ^a^
Security	The ways that participants’ OSA experiences had impacted their feelings of safety.	Anonymity Physical security	Related to ability to hide own identity online. Related to a lack of potential sexual health consequences.	“I may be gay for a while, all other times I am hiding it”. “It allows me to explore sexual fantasies without engaging in behaviors that could be harmful to my physical and mental health”.	Privacy threat Technology threat	Related to privacy threats and personal exploitation. Related to security of the technology itself.	“He took pictures and was going to send them around”. “Your computer can get viruses on suspicious sites!” ^b^

^a^ Quotation was translated from German to English. ^b^ Quotation was translated from Swedish to English.

## Data Availability

The data presented in this study are available on request from the corresponding author. The data are not publicly available due to ethical limitations when using qualitative data.
